# Scrotoschisis: a case report

**DOI:** 10.1186/s13256-017-1427-8

**Published:** 2017-09-13

**Authors:** Souleymane Sidibe, Maxime Coulibaly, Salman Ghazwani

**Affiliations:** 1 Department of General Surgery, Tambacounda Regional Hospital, Tambacounda, Senegal; 2 Department of Anaesthesia, Tambacounda Regional Hospital, Tambacounda, Senegal; 3 Department of Pediatric Surgery, Tambacounda Regional Hospital, Tambacounda, Senegal; 4 Jazan University, P.O. Box 114, Jazan, Postal Code 45 142 Saudi Arabia

**Keywords:** Testicular exstrophy, Scrotal wall abnormality, Orchidopexy

## Abstract

**Background:**

Scrotoschisis is a rare congenital anomaly of the scrotal wall with idiopathic etiology and unknown prevalence. This pathology is extremely rare. We report a new case and review the literature for relevant data.

**Case presentation:**

A 3-day-old full-term baby boy of African ethnicity, who had a homebirth, with birth weight of 2.7 kg presented to our emergency department with exteriorization of left testis; after clinical examination and proper investigations the diagnosis was scrotoschisis. Surgical treatment was performed by primary closure with excellent follow-up. We reviewed the literature to elaborate on the etiology of this pathology and its management.

**Conclusions:**

Scrotoschisis is a rare congenital anomaly affecting healthy babies. Early management is substantial. Further studies are recommended to learn more about the etiology and long-term results, including the effect on the fertility.

## Background

Scrotoschisis is a rare congenital anomaly of the scrotal wall with idiopathic etiology and unknown prevalence. This pathology is extremely rare. In this anomaly, the testis eviscerates through an opening high on the anterior wall of the scrotum [1]. The exact birth prevalence of scrotoschisis is unknown. We report the 17th case in the literature. In this case report, we highlighted etiology hypotheses and management considerations.

## Case presentation

**Fig. 1:**
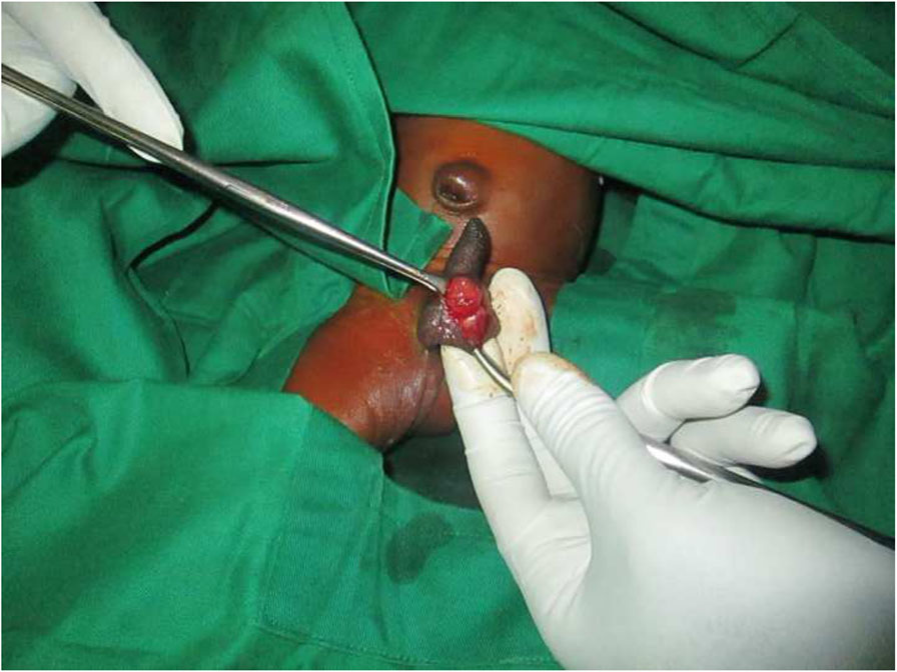
Exteriorizing of the left testis

A 3-day full-term baby boy of African ethnicity presented to our emergency department with exteriorization of left testis from his left hemi-scrotum. He was born at home at 41 weeks of gestational age by normal vaginal delivery: birth weight. 2.7 kg, birth length of 44 cm, and head circumference of 46 cm. His 27-year-old mother, primigravida, is a farmer without a particular past or medical history: there is no family history of the same pathology and no history of consanguinity. An examination revealed no relevant findings for his other organs. There was neither antenatal nor ultrasonography follow-up. A physical examination was remarkable for left scrotal anterior wall defect of 1.5 × 2 cm with normal aspect of inguino-genital region. His left testis was exteriorized within its coverage of tunica vaginalis, mildly edematous with a thin fine exudative inflammatory membrane but with normal vascular appearance. All blood analyses and urine examinations were within normal range. Broad-spectrum antibiotics (ceftriaxone) were started on his arrival at our emergency unit. Under general anesthesia, surgical debridement and scrotal defect exploration showed complete left scrotoschisis with free and mobile extracorporeal testis, no calcified masses, and no meconium residua (Fig. 1). Orchidopexy and primary skin closure was performed horizontally in two layers with vicryl 4-0. There were good results on follow-up at 1 and 2 months. Testicular ultrasound at 3 months showed two identical testes with normal appearance; there was no follow-up beyond 3 months.

## Discussion

Scrotoschisis is a rare congenital scrotal wall pathology. Its prevalence is unknown. Only 16 cases were reported in the literature [2, 3]. It is a semi-urgent condition which should be managed immediately if it presents with a testicular torsion. There are many hypotheses for its etiology: Failure of differentiation of scrotal mesenchyme layer results in rupture or avascular necrosis of overlying epithelium leading to scrotal wall defect [4]. Other reports mentioned external mechanical compression effect due to arthrogryposis [5], as well as meconium periorchitis [6]. In our case, we have no obvious cause; there is no evidence of meconium residua and neither is there evidence of traumatism. Iatrogenic injury is a potential cause and could be concurrently associated if obstetrical difficulties were encountered during labor, mostly if completed by cesarean section [7]. Broad-spectrum antibiotics should be commenced immediately on diagnosis. During a physical examination, it is fundamental to rule out testicular torsion (due to lack of testicular anatomical attachments). A local aseptic dressing procedure is imperative to avoid possible orchitis or peritonitis in case of communicating patent processus vaginalis. A surgical approach depends on the severity and could be a simple dressing with healing by secondary intention [8] or conventional surgical method in two planes horizontally or vertically after orchidopexy. In our case, we preferred to close in two layers horizontally; the skin defect was mainly horizontal. Postoperative follow-up should not be discontinued until verification of two symmetric, vital, and normally growing testes. Further research is recommended to study the effect of scrotoschisis on fertility.

## Conclusions

Scrotoschisis is a rare congenital anomaly affecting otherwise healthy babies. Early management is substantial. Further studies are recommended to learn more about the etiology and long-term results, including fertility.
